# Galactosylceramidase deficiency and pathological abnormalities in cerebral white matter of Krabbe disease

**DOI:** 10.1016/j.nbd.2022.105862

**Published:** 2022-09-14

**Authors:** Diego Iacono, Shunsuke Koga, Hui Peng, Arulmani Manavalan, Jessica Daiker, Monica Castanedes-Casey, Nicholas B. Martin, Aimee R. Herdt, Michael H. Gelb, Dennis W. Dickson, Chris W. Lee

**Affiliations:** a Biomedical Research Institute of New Jersey (BRInj), Cedar Knolls, NJ, United States of America; b Atlantic Health System, Morristown, NJ, United States of America; c Mid-Atlantic Neonatology Associates (MANA), Morristown, NJ, United States of America; d Department of Neuroscience, Mayo Clinic Florida, Jacksonville, FL, United States of America; e Departments of Chemistry and Biochemistry, University of Washington, Seattle, WA, United States of America

**Keywords:** Krabbe disease, Globoid cell leukodystrophy, Galactosylceramidase, Oligodendrocyte, Psychosine, Cerebral white matter, Demyelination, Neuroinflammation, CD8-positive T lymphocyte, Acid ceramidase

## Abstract

Krabbe Disease (KD) is an autosomal recessive disorder that results from loss-of-function mutations in the *GALC* gene, which encodes lysosomal enzyme galactosylceramidase (GALC). Functional deficiency of GALC is toxic to myelin-producing cells, which leads to progressive demyelination in both the central and peripheral nervous systems. It is hypothesized that accumulation of psychosine, which can only be degraded by GALC, is a primary initiator of pathologic cascades. Despite the central role of GALC in KD pathomechanism, investigations of GALC deficiency at a protein level are largely absent, due in part, to the lack of sensitive antibodies in the field. Leveraging two custom antibodies that can detect GALC at endogenous levels, we demonstrated that GALC protein is predominantly localized to oligodendrocytes in cerebral white matter of an infant brain, consistent with its functional role in myelination. Mature GALC could also be quantitatively detected as a 26 kDa band by western blotting and correlated to enzyme activity in brain tissues. The p.Ile562Thr polymorphic variant, which is over-represented in the KD population, was associated with reduced mature GALC protein and activity. In three infantile KD cases, homozygous null mutations in *GALC* lead to deficiency in total GALC protein and activity. Interestingly, although GALC activity was absent, normal levels of total GALC protein were detected by a sandwich ELISA using our custom antibodies in a later-onset KD brain, which suggests that the assay has the potential to differentiate infantile- and later-onset KD cases. Among the infantile KD cases, we quantified a 5-fold increase in psychosine levels, and observed increased levels of acid ceramidase, a key enzyme for psychosine production, and hyperglycosylated lysosomal-associated membrane protein 1, a marker for lysosomal activation, in periventricular white matter, a major pathological brain region, when compared with age-matched normal controls. While near complete demyelination was observed in these cases, we quantified that an early-infantile case (age of death at 10 months) had about 3-fold increases in both globoid cells, a pathological hallmark for KD, and CD8-positive T lymphocytes, a pathological marker for multiple sclerosis, in the white matter when compared with a slower progressing infantile case (age of death at 21 months), which suggests a positive correlation between clinical severity and neuropathology. Taken together, our findings have advanced the understanding of GALC protein biology in the context of normal and KD brain white matter. We also revealed new neuropathological changes that may provide insights to understand KD pathogenesis.

## 1. Introduction

Globoid cell leukodystrophy or Krabbe disease (KD) (OMIM 245200) is an inherited demyelinating disorder caused by a deficiency of galactosylceramidase (GALC; EC 3.2.1.46), which leads to the accumulation of its substrates galactosylceramide (GalCer) and galactosylsphingosine (also known as psychosine) in myelin-producing cells (Wenger et al., 2019). Given that GalCer is readily converted into psychosine via enzymatic diacylation by acid ceramidase (ASAH1) (Li et al., 2019) and GalCer is abundantly produced during the active myelination phase of early brain development, the loss of GALC activity is thought to lead to toxic accumulation of psychosine, as well as dysfunction and death of oligodendrocytes and Schwann cells. This results in demyelination in both central and peripheral nervous systems in KD (Igisu and Suzuki, 1984; Miyatake and Suzuki, 1972; Svennerholm et al., 1980). Despite the central role of GALC in KD pathomechanism, investigations of GALC deficiency at a protein level are largely absent, due in part, to the lack of sensitive antibodies.

Although several classifications have been proposed for KD (Duffner et al., 2011; Wenger et al., 1997), it can be divided into two subtypes based on the age at symptom onset (Orsini et al., 2000). Infantile KD (before 12 months) is the most common and fatal form of the disease, which clinically presents with irritability, feeding difficulties, loss of vision and hearing, and seizures after a few months of normal development. Later-onset KD (after 12 months) usually has a better prognosis, and its clinical presentations vary.

Genetically, KD is caused by homozygous or compound heterozygous mutations in the human *GALC* gene (Tappino et al., 2010; Wenger et al., 1997), which lead to a dramatic reduction or complete deficit of GALC protein function. Null mutations eliminate normal *GALC* transcripts and all functional GALC protein. By contrast, missense mutations impair GALC function through mechanisms such as protein instability, misfolding, mis-trafficking and catalytic inactivation (Deane et al., 2011). Patients carrying the homozygous null mutation, such as a 30 kb deletion in the *GALC* gene, common in those of European descent (Luzi et al., 1995; Rafi et al., 1995), are likely to develop infantile KD; while those with at least one allele, such as p.Gly286Asp mutation (Furuya et al., 1997), develop later-onset KD.

In general, symptomatic KD patients have ≤10% of normal GALC activity as measured in dried blood spots, leukocytes or cultured skin fibroblasts (Orsini et al., 2016; Wenger et al., 1997). While GALC activity provides a critical index for KD diagnosis, it has also been shown to produce a relatively high false-positive rate in newborn screening. Moreover, GALC activities measured in leukocytes or fibroblasts cannot always differentiate between infantile KD and later-onset KD (Wenger et al., 2021). Diagnostic tests that include measurement of psychosine levels provide a more robust index of lysosomal GALC activity (Guenzel et al., 2020; Langan et al., 2019).

Understanding the relationship between KD genotype and GALC deficiency, psychosine accumulation, demyelination, globoid cell pathology and neuroinflammation is an important step in understanding pathogenesis of KD and eventual treatment for KD. Unfortunately, there are only a few studies that link the genetic, biochemical and neuropathological features in KD. In this study, we provide a comprehensive analyses of postmortem brain samples from infantile-KD and later-onset KD compared with age- and sex-matched normal controls.

## 2. Materials and methods

### 2.1. Brain tissues and genetic analyses

We obtained clinical information and brain tissues from a total of nine patients, including four symptomatic KD (all males) with genetically confirmed GALC mutations, and four age-matched controls (all males). Brain tissues and clinical information were obtained on KD cases and controls from the NIH NeuroBioBank consortium (https://neurobiobank.nih.gov). A normal 54-year-old woman was from the Mayo Clinic brain bank. All specimens were obtained after next-of-kin (or legal representative) signed written consent for their use in research, and all procedures followed approved Institutional Review Board regulations.

At autopsy, one-half of the brain from each subject was fixed in 10% buffered formalin for at least two weeks. Coronal sections were grossly and microscopically examined following standard neuropathologic assessment at University of Maryland Brain Tissue Bank (UMBTB), Baltimore, MD or Sepulveda Research Corporation, North Hills, CA. The other half of the brain was fresh-frozen. Fresh-frozen tissue samples from periventricular white matter (PVWM) and formalin-fixed tissue samples from frontal cortex, PVWM, Brodmann Area 24 (BA24), corpus callosum (CC), and hippocampus were obtained for each case.

All cases were had genetic confirmation of KD-related pathogenic *GALC* gene mutations and all four KD cases were determined to carry previously identified pathogenic mutations in *GALC* gene. None of the controls were carriers of any pathogenic *GALC* mutation (See Table 1). The genetic analyses were performed by Genewiz (Plainfield, NJ) using genomic DNA extracted from the fresh-frozen PVWM of each KD and control case. Based on the available clinical history, medical records, and gross pathological findings, none of the control subjects had clinical or neuropathological evidence of cerebrovascular disease, neurodegenerative illnesses or any other major neurological disorder.

### 2.2. Neurohistology procedures

Tissue-blocks were processed by an automated tissue-processor (Tissue-Tek V.I.P. 1000 Vacuum Infiltration Processor, Ames Division, Miles Laboratories, Inc. IN, USA) with standard histology protocols. Tissue blocks were then embedded in paraffin and serially cut using a semi-automatic microtome (Leica RM2255, Leica Biosystems, Nussloch, Germany). For each case, a 5 μm-thick tissue section from each tissue block was stained with hematoxylin and eosin and evaluated microscopically at low (2.5× objective) and high (20× objective) magnification, in order to detect possible hypoxic-ischemic lesions, microvascular pathologies, microhemorrhages, tissue rarefaction or other histopathologic abnormalities. A second 5 μm-thick tissue section from each brain region was stained with Luxol fast blue and periodic acid Schiff counterstain (LFB-PAS) to identify possible myelin loss, rarefaction or other myelin abnormalities, as well as confirmation of presence of globoid cells, a typical neuropathological feature of KD.

### 2.3. Immunohistochemistry

Immunohistochemistry (IHC) was performed on paraffin-embedded sections mounted on glass slides. Antigen retrieval was performed by steaming slides in distilled water or Tris-EDTA buffer pH 9 for 30 min after deparaffinization in xylene and reagent alcohol. All IHC was conducted using IHC Autostainer 480S (ThermoFisher Scientific Inc., Waltham, MA) and DAKO EnVision^™^ + reagents (Dako, Carpinteria, CA) with 3,3′-diaminobenzidine as the chromogen (Dako, Carpinteria, CA). Sections of the BA24, hippocampus with adjacent cortex, PVWM and CC were immunostained with antibodies against GALC protein (CL13.1, 1:5000, mouse monoclonal), myelin basic protein (MAB386, 1:50, rat monoclonal, MilliporeSigma, Burlington, MA), ASAH1 protein (ABN468, 1:100, rabbit polyclonal, MilliporeSigma), LAMP1 protein (E-5, 1:50, mouse monoclonal, Santa Cruz Biotechology, Dallax, TX), activated microglia (CD68) (1:1000, mouse monoclonal, DAKO), astrocytes (GFAP) (GA-5; mouse monoclonal; 1:5000; BioGenex, Fremont, CA), CD4 T-cells (mouse monoclonal, 1:50, DAKO) and CD8 T-cells (mouse monoclonal, 1:500, DAKO). Immunostained slides were counterstained with hematoxylin and coverslipped.

### 2.4. Quantitative digital image analysis

To obtain whole slide images, all immunostained slides and LFB-PAS stained slides were scanned at 20× or 40× magnification using the ScanScopeXT (Leica Biosystems). The regions of interest (i.e., white matter of BA24, PVWM, corpus callosum, and white matter of the parahippocampal gyrus (PHG)) were manually annotated using Aperio ImageScope version 12.4.2.7000 (Leica Biosystems). A custom-designed color deconvolution algorithm was applied to detect immunopositive pixels and to calculate a percent ratio of the area of immunoreactive pixels to the total area of the annotated region (Koga et al., 2018). We also quantified myelinated fibers on LFB-PAS stained slides using a custom-designed color deconvolution algorithm.

### 2.5. Immunofluorescence double-staining

Immunofluorescence staining was performed on sections of the PVWM of Case 2, Case 7, and the adult control brain. Deparaffinized and rehydrated sections were steamed in Citrate buffer (Dako) for 30 min and were blocked with Protein Block plus Serum Free (DAKO) for 1 h. Sections were incubated with anti-GALC antibody (1:1000) and anti-OLIG2 (1:200; ab109186; Abcam, Waltham, MA) antibody diluted in Antibody Diluent with Background-Reducing Components (DAKO) overnight at 4 °C. Sections were washed three times with 1xPBS at room temperature and then incubated with secondary antibodies Alexa Fluor 488 and 568 (1:500, Thermo Fisher Scientific, Inc.) diluted with Antibody Diluent with Background-Reducing Components for 1.5 h at room temperature. Sections were washed three times with 1xPBS at room temperature, incubated with 1% Sudan Black for 2 min, washed with distilled water, and mounted with Vectashield mounting media containing DAPI (Vector Laboratories, Newark, CA).

### 2.6. Western blotting

Thawed fresh-frozen tissue was homogenized in M-Per lysis buffer (ThermoFisher Scientific) supplemented with protease inhibitors. After centrifugation, the protein concentration of the supernatant was determined by BCA assay for normalization, and 30 μg of total protein was resolved by SDS-PAGE with 10–20% Tris-glycine gel. Gel proteins were transferred to PVDF membranes by Criterion blotter (Bio-Rad, Hercules, CA) according to manufacturer’s instruction. The protein blot was blocked in 5% non-fat milk for 1 h at room temperature followed by incubation with primary antibody for 16–18 h at 4 °C. The blot was then probed with HRP-conjugated secondary antibody for 1 h at room temperature, followed by signal development using the Immobilon Western Chemiluminescent HRP substrate (MilliporeSigma). WB images were obtained by the Image-quant LAS 4000 mini system. Primary antibodies for WB analysis include anti-GALC (CL13.1, 1:4000) (Lee et al., 2007); anti-MBP (MAB386, 1:500, MilliporeSigma); anti-Lamp1 (E-5, 1:1000, Santa Cruz Biotechnology); anti-ASAH1 antibody (ABN468, 1:2000, MilliporeSigma); anti-GAPDH (1:100,000, Proteintech, Rosemount, IL).

### 2.7. GALC activity assay

GALC enzymatic activity in tissue supernatant was measured by a fluorescence substrate turnover assay previously described in (Wiederschain et al., 1992). Briefly, 10–20 μg total protein was added to a reaction cocktail containing 0.1 M citric acid, 0.2 M sodium phosphate, 7 mg/ml sodium taurocholate, 2 mg/ml oleic acid and 0.5 mg/ml 6-hexadecanoylamino-4-methylumbelliferyl-β-D-galactopyranoside (Biosynth Carbosynth Inc., Newbury, U.K.) at pH 4.5. The reaction was incubated for 4–6 h at 37 °C and stopped by adding 2 volumes of 0.1 M glycine/ 0.1 M sodium hydroxide solution and 4 volumes of absolute ethanol. The concentration of reaction product was determined on a fluorescence plate reader (ex 385 nm/ em 450 nm). A 4-methylumbelliferone standard curve was ran in parallel for calculation of absolute GALC activity expressed in nmol/h/mg protein.

### 2.8. GALC enzyme-linked immunosorbent assay (ELISA)

Twenty microgram of total protein from tissue supernatant diluted in sample buffer (PBS with 1% BSA) was added to a Nunc Maxisorp^™^ 96-well plate pre-coated with 3 mg/ml anti-GALC antibody, CL1021AP (Lee et al., 2010) and pre-blocked in sample buffer. Standard curve samples were prepared by diluting recombinant human GALC protein (R&D Systems) in sample buffer to 0.8, 1.6, 3.2, 6.3, 12.5, 25 and 50 ng/ml. Samples and standards were incubated on the plate for 2 h. Anti-GALC antibody, CL13.1, followed by anti-mouse IgG1-HRP secondary antibody, both diluted in sample buffer to 0.5 mg/ml, were added to the plate and each incubated for an hour. For signal development, TMB substrate was added to the plate, incubated for 30 min. Diluted sulfuric acid (0.16 M) was added to stop the reaction. The plate was read on the SpectraMax Plus 384 microplate reader (450 nm). All procedures except for antibody coating were performed at room temperature. Plate washing using PBS with 0.05% Tween-20 was performed in-between blocking, sample addition and antibody addition steps. GALC protein amount (nanogram per milligram protein) was calculated by interpolation of absorbance values to a normalized standard curve.

### 2.9. LAMP1 protein deglycosylation

Deglycosylation reaction was performed following instructions from the PNGase F kit purchased from New England Biolabs, Ipswich, MA (P0705). Briefly, 20 μg of total protein from tissue supernatant was first denatured in 1× denaturation buffer at 100 °C for 10 min. Then the denatured protein was deglycosylated with 500 units of PNGsae F in 1× Glyco-buffer and 1% NP-40 at 37 °C for an hour. Negative control reaction without the addition of PNGase F was prepared for each sample for side-by-side comparison of glycosylation status. The reaction was quenched by SDS sample buffer and reducing reagent mix, then resolved by SDS-PAGE with 10–20% Tris-glycine gels for subsequent LAMP1 detection by Western blotting mentioned previously.

### 2.10. Psychosine measurements

Frozen brain tissue was homogenized in ice cold physiological saline. Protein concentration of the total homogenates were determined by BCA. A portion of the homogenate was shipped to Dr. Gelb’s lab for psychosine analyses. The detailed procedures for sample preparation and UPLC-MS/MS analysis were recently described (Weinstock et al., 2020).

## 3. Results

### 3.1. GALC genotypes in the KD brain cohort

In this study, we evaluated 3 infantile KD, 1 later-onset KD and 4 age-matched normal brains collected from NeuroBioBank (Table 1). To study the link between disease severity, disease duration and biochemical changes, normal control cases (#1 through #4) had increasing age at death (6.7 months, 18 months, 21 months and 40 years) and KD cases (#5 through #8) also had increasing age at death (10 months, 17 months, 21 months and 40 years).

DNA sequencing of the coding region of the *GALC* gene showed that 2 infantile KD cases (#6 and #7) were homozygous for the European 30 kb deletion. One infantile KD case (#5) carried a 30 kb deletion allele, but no known pathogenic mutation was identified on the second allele, which suggests that this patient may have a non-coding mutation. The later-onset KD case (#8) had compound heterozygous mutations at p. Ser23Ter and p.Met117Leu on p.Ile562Thr polymorphic background. The p.Ile562Thr variant is known to impair GALC activity, and is a common cis-polymorphism in KD (Shin et al., 2016; Spratley et al., 2016). No known KD-related mutations were identified in the controls; however, cases #1 and #4 were homozygous for the p.Ile562Thr variant; and cases #2 and #3 were heterozygous for the p.Ile562Thr variant. Additionally, case #3 carried 2 other polymorphisms (p. Ala21Pro and p.Asp248Asn) on the other allele that likely attenuate GALC activity (Saavedra-Matiz et al., 2016).

### 3.2. GALC genotypes, GALC activity and GALC protein levels in KD brains

**Fig. 1.**
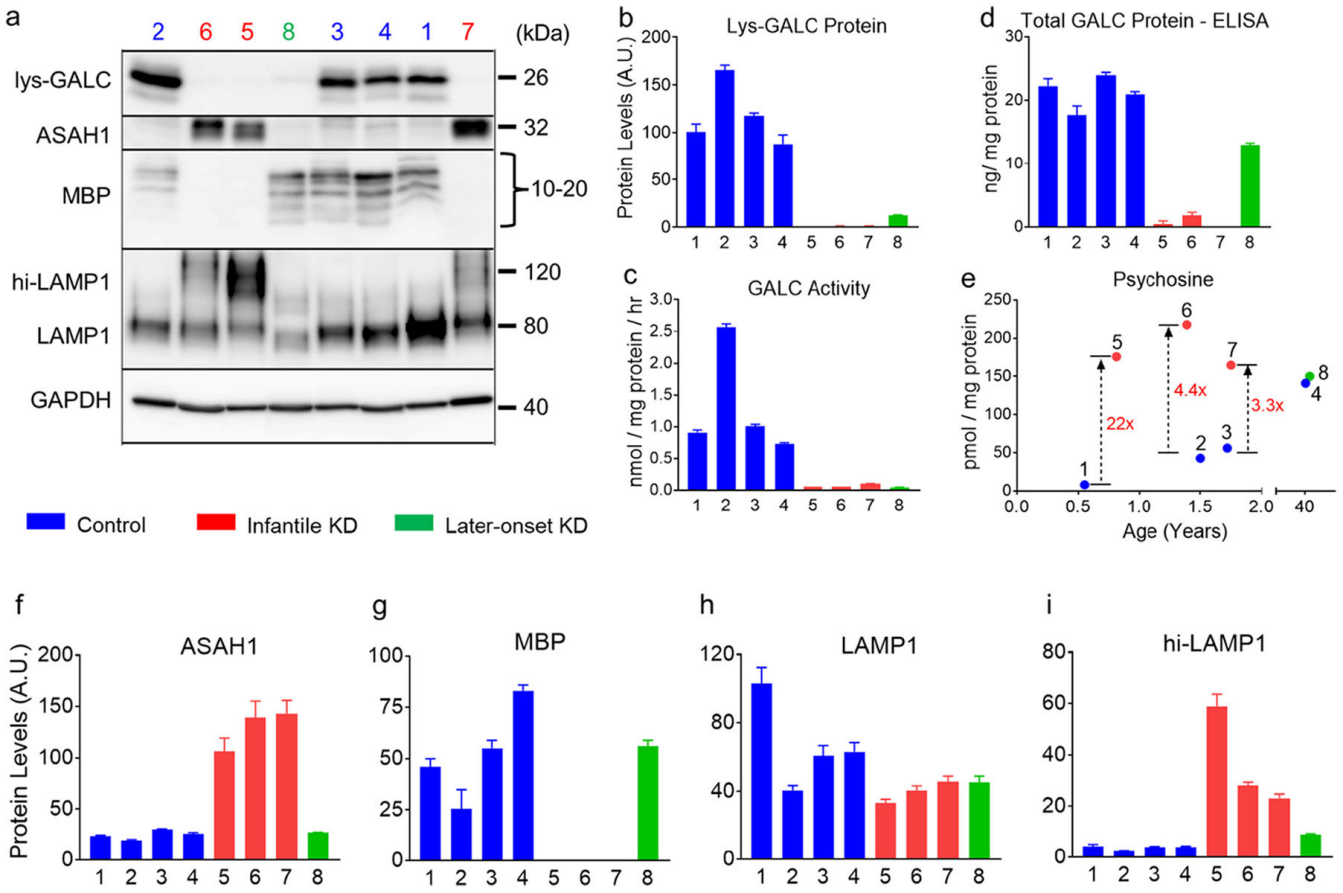
Biochemical abnormalities in the periventricular white matter of KD. Homogenates of periventricular white matter (PVWM) from 4 KD and 4 age-matched normal control cases are analyzed for lysosomal GALC protein (Lys-GALC), mature β-subunit of acid ceramidase (ASAH1), myelin basic protein (MBP), lysosome-associated membrane protein 1 (LAMP1) and glyceraldehyde-3-phosphate dehydrogenase (GAPDH) by Western blotting (a). Protein levels are quantified by densitometric measurement of target band intensity normalized to GAPDH levels. Quantification data of Lys-GALC, ASAH1, MBP, LAMP1 and hyperglycosylated LAMP1 (hi-LAMP1) are shown in panel b, f, g, h and i, respectively. The same samples are also analyzed for GALC activity (c), total GALC protein levels (d), and psychosine levels (e). Increased psychosine levels (fold change) in the three infantile KD cases compared to age-matched controls are also annotated (e). Error bars represent standard error mean of triplicate samples.

To examine how the *GALC* genotypes translate into GALC protein phenotypes, we performed activity assay and Western blotting to quantify the function and protein levels of mature, lysosomal GALC (Lys-GALC) in the PVWM, a major pathological site in KD (Fig. 1a-c). Interestingly, GALC activity was similar in control cases #1, #3 and #4, which all carry at least 2 copies of polymorphic variants on the *GALC* gene. In contrast, GALC activity was 2.5 times higher in control case #2, which only had one copy of the p.Ile562Thr polymorphic variant (Fig. 1c, cases #1–4). The trend was consistent with Lys-GALC protein levels. Control case #2 had 62% more Lys-GALC compared to the mean of the other control cases (Fig. 1b, cases #1–4). Our results suggest that polymorphic variants impair GALC activity by causing reduction in Lys-GALC protein levels. In KD, GALC activity for all cases was below 0.1 nmol/mg/h, which is <5% of the activity observed in control brains, regardless of genotype (Fig. 1c, cases #5–8). Lys-GALC protein levels was low (~14% of control), but detectable in the later-onset case (#8). Lys-GALC protein was undetectable in all infantile KD cases (Fig. 1b, #5–7). The loss of GALC activity in KD was associated with absence of Lys-GALC protein in PVWM.

We also examined total GALC protein levels in PVWM by a sandwich enzyme-link immunosorbent assay (ELISA) using our custom antibodies CL13.1 and CL1021AP. As shown in Fig. 1d, similar levels of total GALC protein (i.e., approximately 20 ng/mg) were detected in all control cases. Interestingly, while all infantile KD cases had almost no detectable GALC protein due to the homozygous null *GALC* mutations, the later-onset KD (case #8) had about 60% of control levels (i.e., 13 ng/mg), consistent with a heterozygous genotype. Our results show that GALC protein quantification in patient samples can provide information of *GALC* mutation status.

### 3.3. Disease severity-dependent increase in psychosine accumulation in KD

Psychosine is an important biomarker for diagnosis and newborn screening in KD. To determine how brain psychosine levels are related to disease severity, we compared psychosine levels in PVWM of age-matched controls and compared them to KD (Fig. 1e). The most severe KD case (#5, age of death of 294 days) had a psychosine level of 176 pmol/mg, which was 22 times higher than control case (#1, age of death 202 days). Psychosine levels in KD cases #6 and #7 (age of death 16.7 months and 21 months) were 4.4 times (218 pmol/mg) and 3.3 times (165 pmol/mg) higher than age-matched controls (mean = 49.5 pmol/mg). Overall, an average of 5.2 times increase in psychosine levels was observed in the infantile KD cases (mean of #5, #6 and #7 = 186 pmol/mg) compared with the controls (mean of #1, #2 and #3 = 35.6 pmol/mg). Of note, brain psychosine levels in the late-onset case (#8) were only slightly higher than age-matched controls (#4) (150 pmol/mg vs. 141 pmol/mg, respectively). Our data show a trend of positive correlation between psychosine levels and disease severity. Moreover, increases in psychosine can be pronounced in KD patients with very early age of death. By contrast, brain psychosine levels in normal controls increases with age (8, 42.8, 56.1 and 141.1 pmol/mg, respectively), emphasizing the necessity of using age-matched controls in studies of psychosine in KD.

### 3.4. Severe demyelination in infantile KD brain tissues

To examine biochemical changes associated with demyelination in KD, we analyzed levels of myelin basic protein (MBP), a major component of myelin sheath. We detected normal MBP species (10 to 20 kDa) in all control cases with a specific and well-characterized MBP antibody (Western blot, Fig. 1a). MBP was completely absent in all infantile KD (i. e., cases #5, #6, #7), while later-onset KD (case #8) had normal MBP (Fig. 1g). Consistent with MBP reduction in infantile KD, we also observed an almost complete disappearance of myelin in infantile cases (#5 and #7) in multiple regions with LFB-PAS stained tissue sections (Fig. 5a-c). Using digital imaging analysis, we found a 99% reduction in myelin signal in these KD cases compared with control case #2 in PVWM, BA24 subcortical white matter, corpus callosum, and parahippocampal white matter.

### 3.5. Aberrant increase of ASAH1 and hyper-glycosylated LAMP1 protein in infantile KD brain tissues

**Fig. 2.**
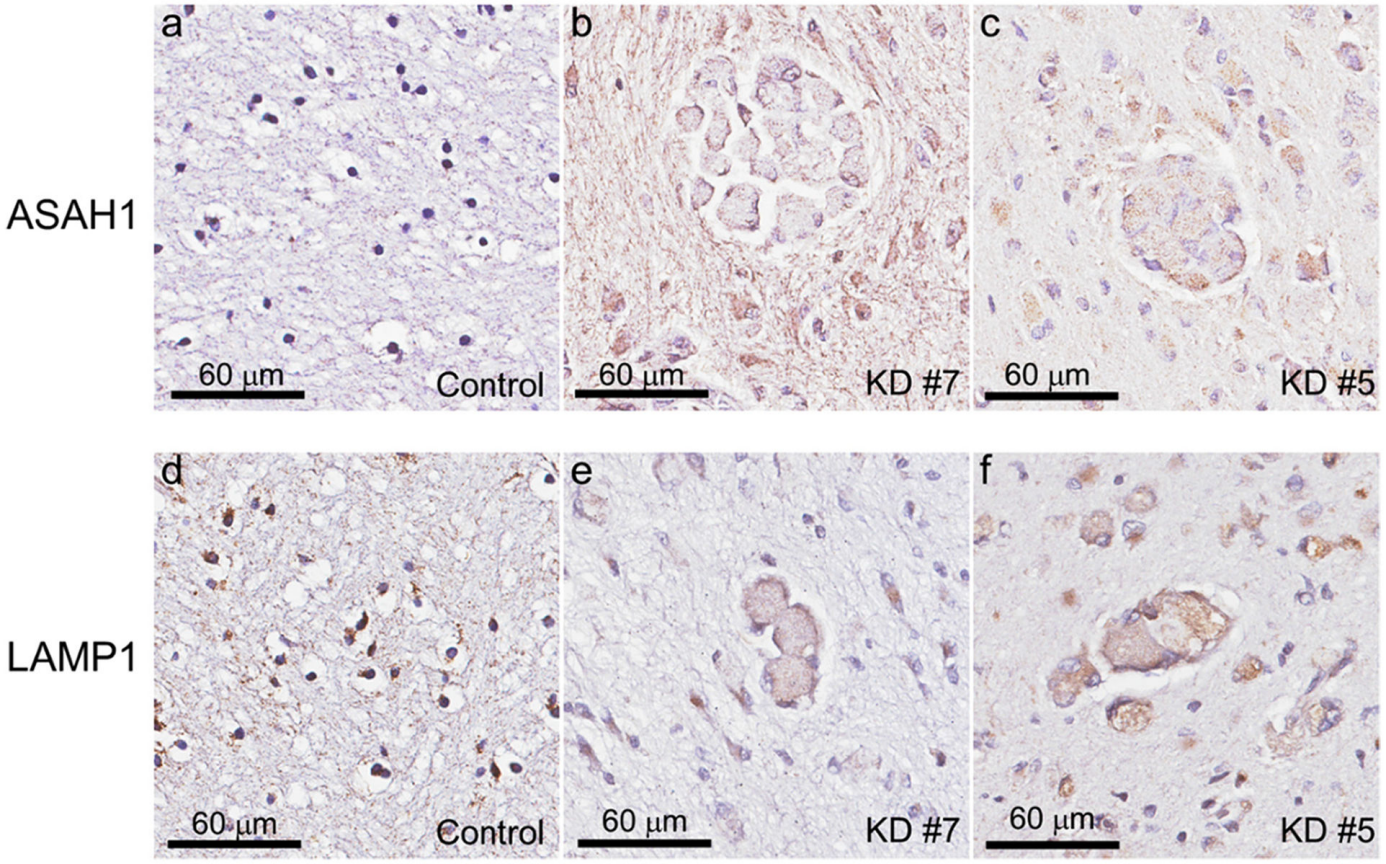
ASAH1 and LAMP1 immunoreactivity in PVWM of infantile KD brains. Immunoreactivity of ASAH1 (b, c) and LAMP1 (e, f) are observed in PVWM of infantile KD (cases #5 and #7) compared with the normal control case #2 (a, d).

Given the enzymatic role of ASAH1 in production of psychosine, we sought to examine levels of ASAH1 protein in KD. It has previously been shown that ASAH1 undergoes autocleavage into the α- and β-subunits upon maturation in the lysosome (Gebai et al., 2018). We found that protein levels of ASAH1 β-subunit (32 kDa) were significantly increased (5-fold) in all three infantile KD cases compared to controls and the later-onset KD case (Western blot, Fig. 1a and f). Similarly, ASAH1 protein expression was observed in PVWM immune cells of infantile KD cases (#5 and #7; Fig. 2b, c). An insignificant amount of ASAH1 signal was detected in controls (case #2) (Fig. 2a). These results suggest that increased psychosine levels in infantile KD cases may not only arise due to a decrease in GALC catabolism, but also may be the result of increase production by ASAH1.

**Fig. 3.**
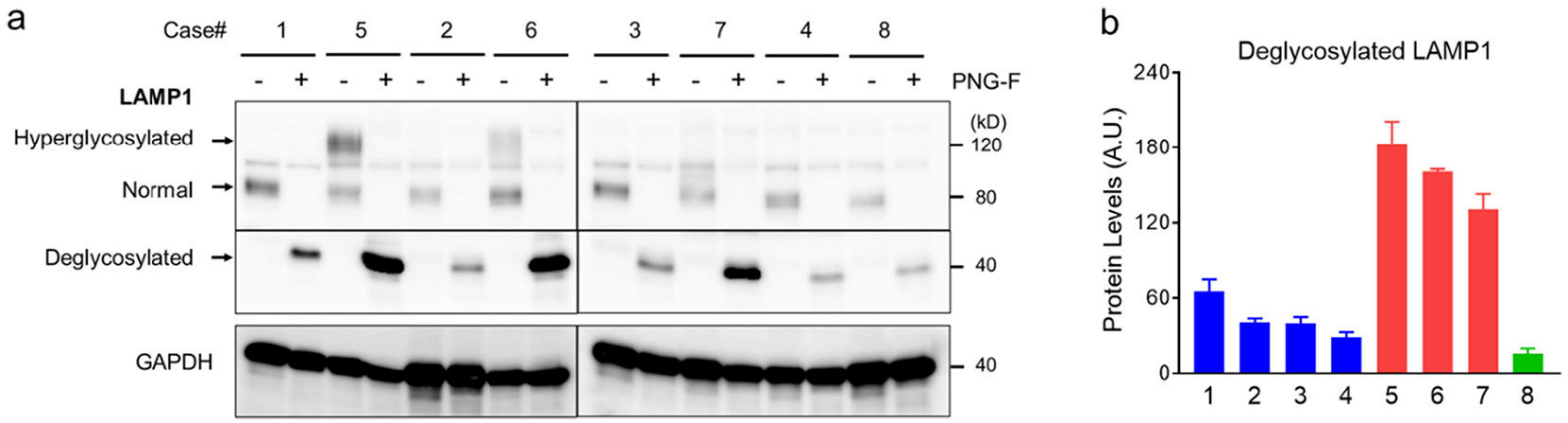
Increased LAMP1 protein levels in infantile KD. (a) PVWM lysate is treated with and without PNGase F to remove glycosylation from total proteins then analyzed for LAMP1 protein species by Western blotting. The molecular weights of normal form (80 kDa) and hyperglycosylated form (120 kDa) of LAMP1 are reduced and merge after deglycosylation with PNGase F (40 kDa). (b) The deglycosylated LAMP1 protein is quantified by densitometric measurement of target band intensity normalized to GAPDH levels. Error bars represent standard error mean of triplicate samples.

Lysosome-associated membrane protein 1 (LAMP1) is one of the most abundant membrane proteins in lysosomes and is commonly used to study the role of lysosomal activation in neuroinflammation. We identified two forms of LAMP1 in our case series. A smaller, 80 kDa species was observed in control cases and in the late-onset KD case, while infantile KD cases expressed the 80 kDa and a larger 120 kDa species (Fig. 1a). The levels of the 120 kDa LAMP1 species were trended positively in correlation with disease severity, such that the most severe case (#5) had about twice the protein levels compared with less severe infantile KD cases (#6 and #7) (Fig. 1i). We suspect that the larger LAMP1 species (120 kDa) may be caused by hyper-glycosylation, as previously observed in in brain of Niemann-Pick disease type C1 (NPC1) from mouse models and patients (Cawley et al., 2020). To validate this hypothesis, we performed an in vitro deglycosylation reaction, prior to Western blot analysis, to determine the glycosylation status of the larger LAMP1 species. LAMP1 signal collapsed into single band at 40 kDa in both control and KD samples indicating that the 120 kDa species was a hyperglycosylated form (Fig. 3a). In addition, levels of deglycosylated LAMP1 protein were increased in all infantile KD cases by about 3-fold when compared with age-matched controls, indicating an increase in total LAMP1 levels (Fig. 3b). Histologically, LAMP1 was detected in the form of puncta staining in the oligodendrocytes of control case #2 (Fig. 2d). LAMP1 signal was apparently detected in PVWM globoid cells of infantile KD cases (#7 and #5, Fig. 2e, f).

### 3.6. IHC and digital quantification of GALC protein in normal infant and adult brain white matter

**Fig. 4.**
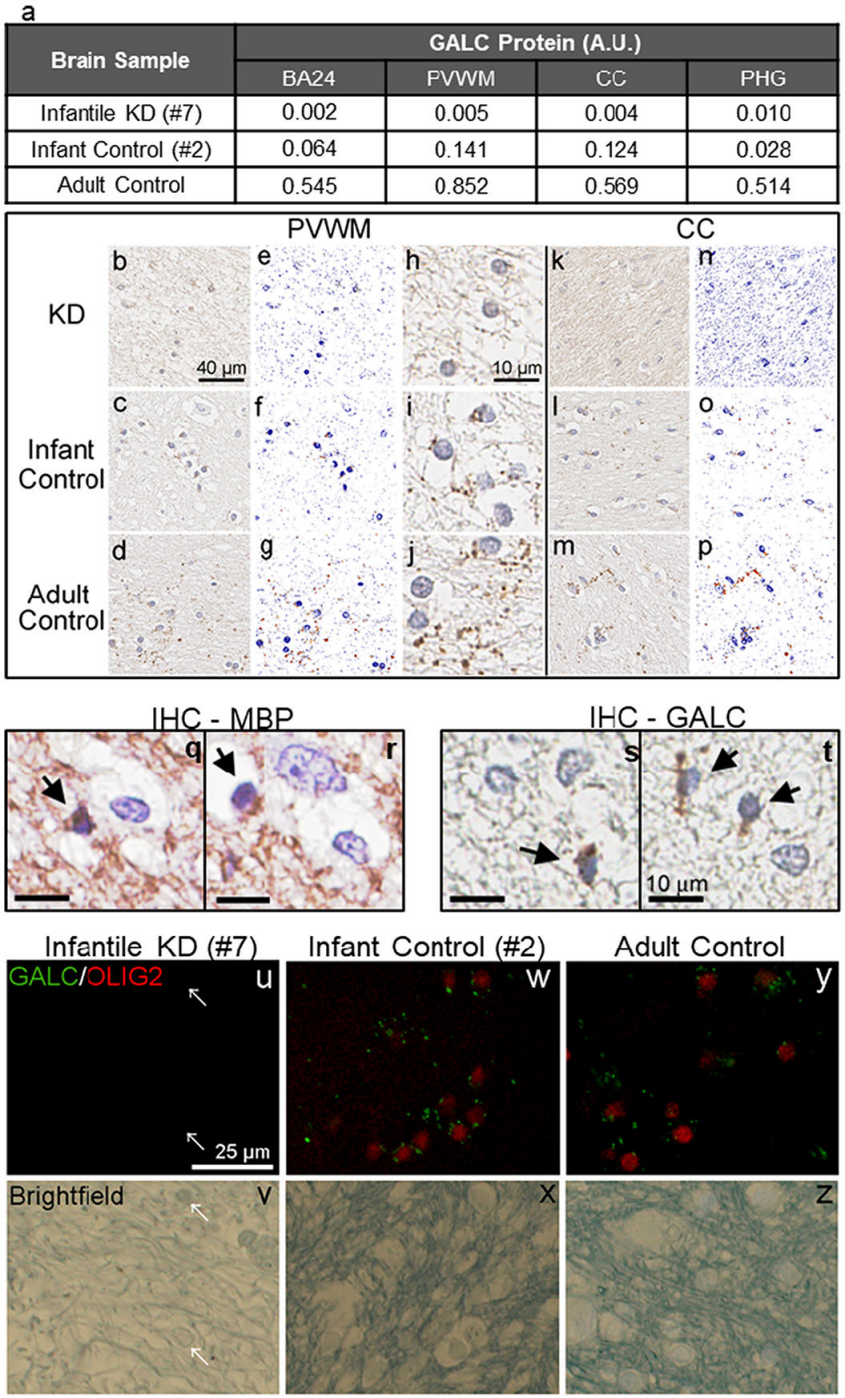
Quantitative Immunohistochemistry of GALC in cerebral white matter and GALC localization in oligodendrocytes. Subcortical white matter in BA24, PVWM, corpus callosum (CC), and parahippocampal gyrus of a normal infant (case #2) and a normal adult are immunostained with an anti-GALC antibody. Infantile KD (case #7) with homozygous 30 Kb deletion on *GALC* gene is used as a negative control. GALC immunoreactivity is quantified by digital imaging analysis using the Aperio ImageScope software (a). Representative images of GALC immunohistochemistry in PVWM (b, c, d) and CC (k, l, m) are shown at 40× magnifications. Representative color deconvoluted images applied for digital quantification in the PVWM and CC are shown in panels e, f, g and panels n, o, p, respectively. Magnification of panels b, c and d are shown in panels h, i and j, respectively. Oligodendrocytes (arrow), characterized by small oval nucleus and dense chromatin, are immunopositive for MBP (q, r) and GALC (s, t). Representative images of immunofluorescence double-staining of GALC (green) and OLIG2 (red), as well as the matched brightfield in the infantile KD case #7 (u, v), infant control case #2 (w, x) and adult control (y, z) in the PVWM. The brightfield image shows a few remaining cells in the white matter (arrows), but there are no signals of GALC and OLIG2 in the KD case #7 (u, v). In infantile and adult control cases, GALC signals are mainly observed in OLIG2-positive oligodendrocytes (w, y).

GALC is a lysosomal enzyme essential for sphingolipid metabolism in myelin-producing cells, including oligodendrocytes and Schwann cells. Enzymatically, it hydrolyzes the galactose ester bond on galactosylceramide and psychosine to form ceramide and sphingosine, respectively. This is part of the salvage pathway in lysosomes (Paciotti et al., 2020), which plays a key role in myelination and myelin maintenance. GALC functional deficiency has been thought to lead to progressive demyelination in the central and peripheral nervous systems (Feltri et al., 2021; Miyatake and Suzuki, 1972). Haploinsufficiency of GALC also causes impaired remyelination (Scott-Hewitt et al., 2017). The cellular localization of endogenous GALC protein has only recently been demonstrated in Schwann cells and oligodendrocytes of wild-type mice using the CL1021AP antibody (Weinstock et al., 2020). Given the important role of GALC in myelin biology and KD pathogenesis, we performed IHC to detect GALC protein in normal human brain tissue. The infantile KD case #7, which carries the homozygous 30 kb deletion and was demonstrated to have only baseline activity and GALC protein levels (Fig. 1b, c, d), was used as a negative control for GALC-IHC. Immunohistochemistry with CL13.1 showed puncta in the white matter of various brain regions in normal infant (case #2) and in a normal adult control, but not in infantile KD (case #7) (Fig. 4). The puncta signal resembled vesicular structures clustering around oligodendrocytes and sparsely distributed along axonal fibers in the white matter (Fig. 4i, j, s, t and Supp. Fig. 1). The identity of oligodendrocytes was confirmed morphologically by their small oval-shaped nucleus with dense chromatin and with MBP immunoreactivity (Fig. 4q, r). GALC localization in oligodendrocytes was also confirmed by double immunofluorescence staining of GALC and oligodendrocyte transcription factor 2 (OLIG2), an oligodendrocyte-specific marker, in the PVWM of both the normal infant (#2) and adult controls (Fig. 4w, y). Interestingly, the GALC puncta were minimally detected in neuronal cells in BA24 and PHG regions of the infant brain (Supp. Fig. 1d, h). Upon quantification, GALC protein levels were highest levels in PVWM and CC, followed by BA24 white matter, and lowest in parahippocampal white matter in the normal infant brain. The normal adult brain had much higher GALC levels in all white matter regions examined (Fig. 4a, j, m).

### 3.7. Disease-dependent increase in globoid cells, astrogliosis and CD8+ T lymphocytes in white matter of KD

Neuroinflammation is thought to play a role in the pathogenesis of KD (Feltri et al., 2021; Potter and Petryniak, 2016). Activated microglia, along with infiltrating macrophages, and activated astrocytes are common pathological features in white matter and peripheral nerves of KD. Globoid cells, which are multinucleated microglia and macrophages (Supp. Fig. 2), are a pathological hallmark of KD, particularly in infantile cases.

**Fig. 5.**
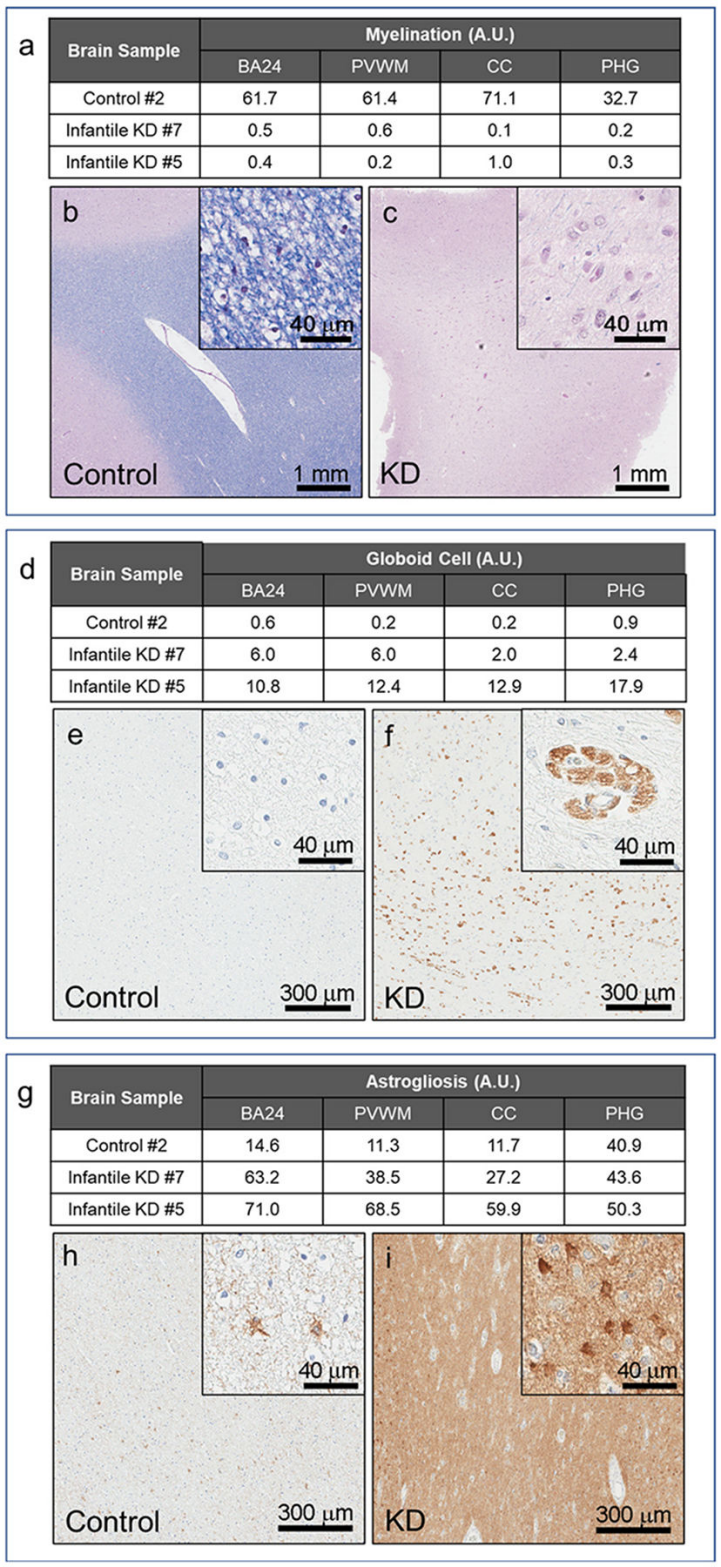
Quantitative neuropathology in the white matter regions of infantile KD. Neuropathological features of infantile KD are illustrated in two cases (cases #5 and #7) compared to an age-matched normal control (case #2). White matter regions (BA24, PVWM, CC and PHG) are analyzed for levels of myelination, globoid cells and astrogliosis with Luxol fast blue stains (a-c), CD68 immunohistochemistry (d-f) and GFAP immunohistochemistry (g-i), respectively. Representative images of LFB-PAS stain (b, c), CD68 (e, f) and GFAP (h, i) in BA24 white matter region of control and KD cases are shown with results of quantification (a, d, g).

To understand the role of neuroinflammation in KD, we analyzed the extent and distribution of neuroinflammatory markers, including CD68-positive globoid cells and glial fibrillary acidic protein (GFAP)-positive astrocytes, in white matter of infantile KD. Globoid cells were found throughout PVWM, as well as white matter in subcortical BA24, PHG and CC in infantile KD (cases #7 and #5) (Fig. 5d-f). Morphologically, globoid cells were large, multinucleated cells that often appeared in clusters (Fig. 5f). No CD68-positive cells were detected in the infant control case (#2) (Fig. 5d, e). Globoid cells were more frequent in the most severe infantile KD case (#5) in all the regions studied, with 1.8-fold increase in subcortical BA24, 2.1-fold increase in PVWM, 6.5-fold increase in CC and 7.5-fold increase in PHG compared to the infantile KD case #7 (Fig. 5d, Fig. 7b). Globoid cells were numerous in all white matter regions examined in case #5. Globoid cells were more prominent (37× higher) in subcortical BA24 and PVWM compared with CC and PHG regions in infantile KD case (#7), who had milder pathology (Fig. 5d).

In addition to widespread globoid cells, astrogliosis was prominent in the white matter of KD. GFAP is a specific marker for astrocytes. Although GFAP also labels non-reactive astrocytes, activated reactive astrocytes had elevated GFAP levels that are positively correlated with the extent of astrogliosis. Basal GFAP was detected in infant control case #2 in all white matter regions. While subcortical BA24, PVWM and CC all had similar levels, PHG had about 3.5-fold higher GFAP signal (Fig. 5g, h). Similar to the distribution of globoid cells, astrogliosis was more prominent in severe infantile KD (case #5) in all white matter regions when compared with infantile case #7, although to a less extent than the difference in globoid cells. The largest difference was observed in the CC and PVWM regions, which were 2.2- and 1.8-fold higher, respectively, in case #5 compared with case #7. GFAP was similar in subcortical BA24 and PHG regions of both cases (Fig. 5g, i, Fig. 7c).

**Fig. 6.**
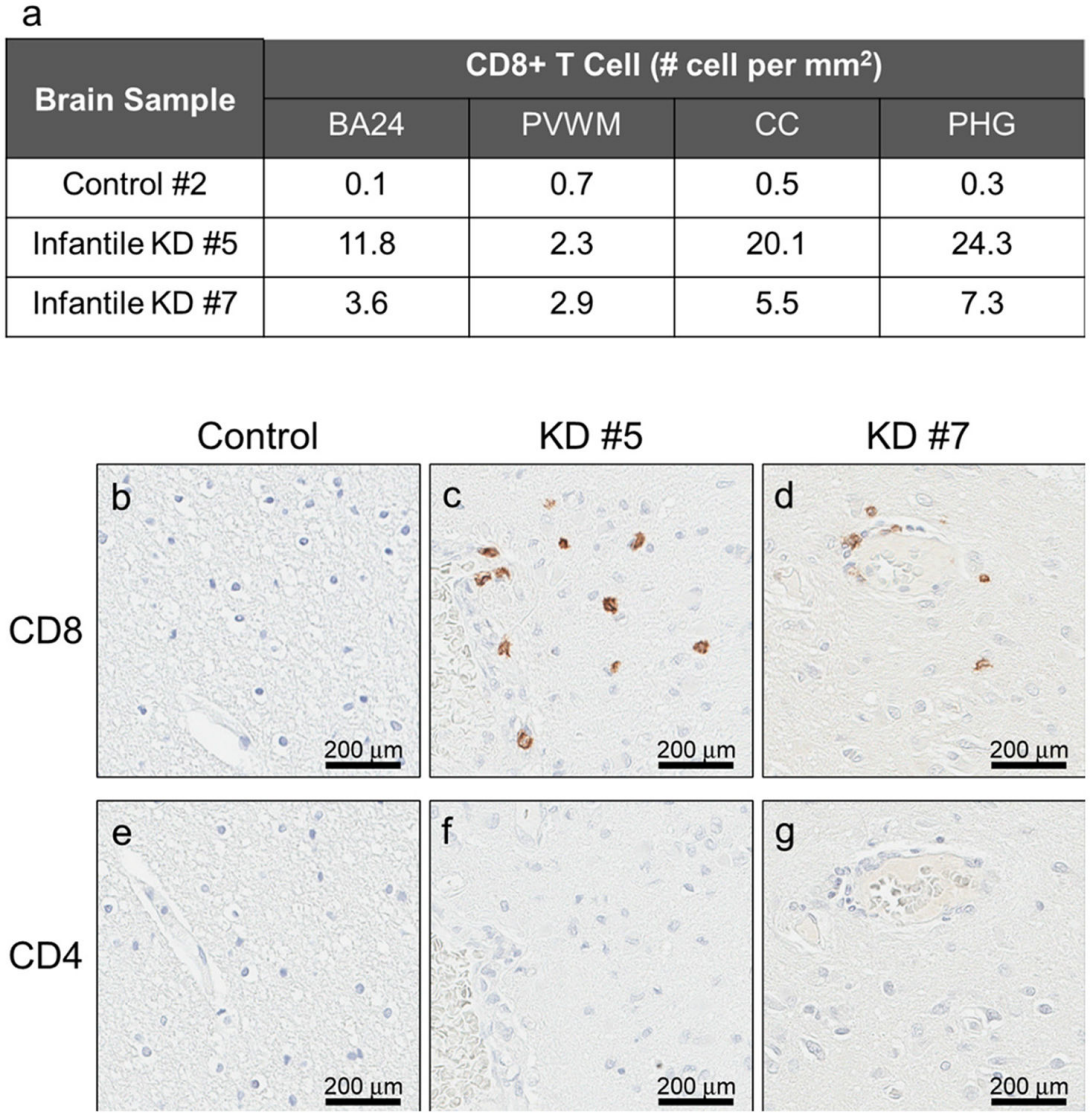
CD8+ T lymphocytes, but not CD4+ T lymphocytes, are present in white matter of infantile KD. White matter in BA24, PVWM, CC and PHG are immunostained for CD4 and CD8 in infantile KD (cases #5 and #7) and a normal infant (case #2). Representative images of CD8 (b, c, d) and CD4 (e, f, g) in BA24. Results of quantification are shown in Table a.

**Fig. 7.**
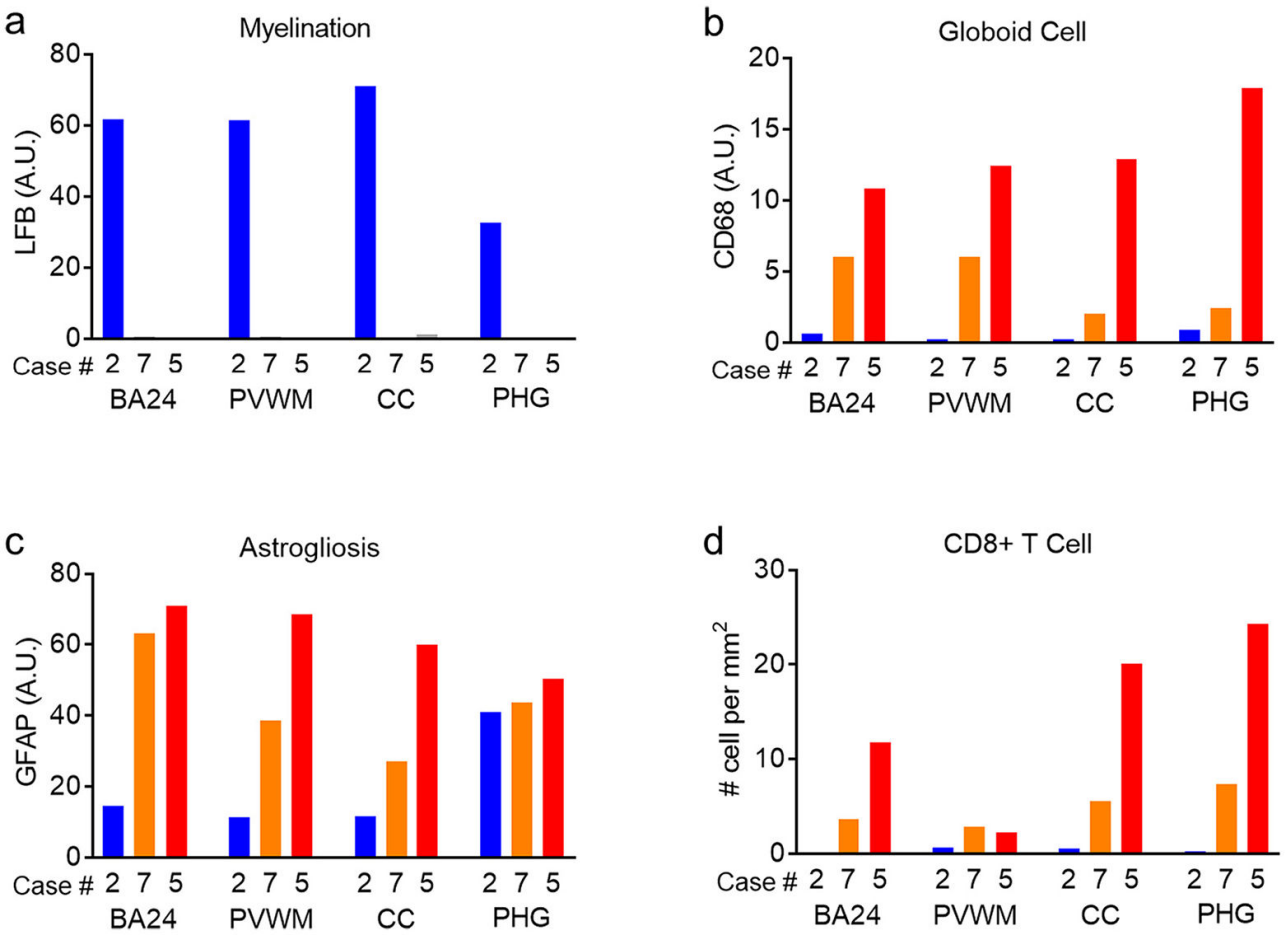
Disease severity-dependent increase of neuropathology in the white matter regions of infantile KD brains. Image quantification results for myelination, globoid cells, astrogliosis and CD8+ T cells in white matter (BA24, PVWM, CC, PHG) of a control (case #2, blue) and of two infantile KD cases with different disease severity (case #7, orange; more severe case #5, red) are plotted in graphical representations.

CD4+ and CD8+ T lymphocytes have been pathologically implicated in patients with demyelination conditions, such as experimental autoimmune encephalomyelitis and multiple sclerosis (Sospedra and Martin, 2005; Wagner et al., 2020). Therefore, we assessed the burden and distribution of CD4+ and CD8+ T lymphocytes in white matter of KD (Fig. 6). Interestingly, CD8+, but not CD4+ T lymphocytes were detected throughout PVWM, BA24 white matter, CC, and PHG white matter of infantile KD (#5 and #7). CD8+ T cells were localized in brain parenchyma, proximal to globoid cells and perivascular spaces (Fig. 6c, d). The number of CD8+ cells was 3- to 4-fold higher in severe infantile KD (#5), except in the PVWM (Fig. 6a, Fig. 7d). Neither CD4+ nor CD8+ T lymphocytes were detected in control case (#2) (Fig. 6b, e).

Overall, our findings describe and quantify well known and new neuroinflammatory features in the end-stage infantile KD. We observed a severity-dependent increase in globoid cells, astrogliosis and CD8+ T cells in various regions of white matter (Fig. 7).

## 4. Discussion

We performed comprehensive biochemical and histopathologic analyses of postmortem brain tissues from KD with different degrees of pathologic severity. To our knowledge, this is the first study that examines GALC deficiency in KD compared with age-matched normal brains with respect to genetics, protein levels, enzyme activity and histology methods. As expected, we found that KD with homozygous null mutation, such as the 30 kb deletion, results in loss of GALC protein expression, with barely background levels of total GALC and Lys-GALC proteins, as well as minimal GALC activity in brain homogenates. Although no KD-related mutation was identified on one allele of case #5, the baseline total GALC protein levels strongly suggests that this patient is a homozygous null mutation carrier. On the other hand, the later-onset KD (case #8) carried a heterozygous missense mutation variant (MMV) (p.Met117Leu) that drives expression of about half GALC protein levels despite the lack of significant differences in GALC activity compared with other infantile KD. These two examples demonstrate that GALC protein ELISA provides an additional method to differentiate homozygous null-*GALC* mutations from *GALC*-MMV genotype, which separates infantile and later-onset KD. Given the time needed for molecular analysis of *GALC* and limited coverage of coding regions with DNA sequencing, GALC protein measurement by ELISA can hasten determination a clinical diagnosis.

One of the important findings of genetics of KD is the role of the p. Ile562Thr pseudo-deficiency variant in *GALC*. It was estimated that 64% to 77% of later-onset KD patients have at least one copy of the variant in cis with a KD-related mutation (Debs et al., 2013). Among 348 infants in a referral group from the New York Newborn Screening program, who had equal or <12% of control GALC activity, the carrier frequency was determined to be 87% (Orsini et al., 2016). Mechanistically, the inheritance of this polymorphic background on a KD-MMV allele attenuates GALC activity, leading to decreased activity sufficient to surpass the threshold needed to drive disease onset (Wenger et al., 2014). For example, the p.Ile562Thr background was found to reduce GALC activity, lysosomal localization and Lys-GALC protein levels in the p. Tyr567Ser variant when expressed in HEK293 cells (Shin et al., 2016). The p.Ile562Thr polymorphic background was also shown to severely reduce secretion and lysosomal localization of GALC protein in carriers of the p.Gly286Asp variant when expressed in Hela cells (Spratley et al., 2016). In the current study, we confirmed a gene dose-dependent effect of the p.Ile562Thr variant in brain tissue. Control cases carrying two copies of the p.Ile562Thr variant (cases #1 and #4) had significantly less Lys-GALC protein and GALC activity in brain homogenates compared to controls carrying only one copy of the variant. Given the prevalence of the p.Ile562Thr polymorphism in KD and the likelihood that this variant impairs GALC function through effects on protein misfolding, development of therapeutic targets, such as pharmacological chaperones that stabilize protein is a potential direction for drug discovery in KD patients carrying the p.Ile562Thr variant.

The case with later-onset KD (case #8) was a Polish patient who had progressive spastic quadriparesis. He was diagnosed with later-onset KD at 13 years of age, and he died at 40 years of age (Grewal et al., 1991; Kolodny et al., 1991). GALC variant expression was studied in COS7 cells to determine the effect of p.Met117Leu and p.Ile562Thr genotype on GALC activity (De Gasperi et al., 1996). The p.Met117Leu and p. Ile562Thr genotype reduced GALC activity by 90%. In the current study, we detected a comparable 94% reduction in GALC activity in brain homogenates of case #8 compared with an age-matched control (case #4). The fact that we were unable to distinguish later-onset KD from infantile KD by GALC activity in brain homogenates is consistent with previous observations in clinical samples, such as leukocytes and skin fibroblasts (Kolodny et al., 1991; Wenger et al., 1974; Young et al., 1972). The failure to distinguish cases with different clinical phenotypes may be due to limitations of analysis of postmortem tissue samples. As mentioned, GALC protein is highly enriched in oligodendrocytes, which are greatly decreased in end-stage KD brains. Heterogeneity of surviving cell populations in autopsy samples may increase variability in GALC activity.

Analysis of non-KD control brain tissues showed age-dependent increases in psychosine in PVWM. Psychosine levels were increased from 8 to 50 pmol/mg protein in samples with 0.6 and 1.6 years of age. This indicates a 6-fold increase in psychosine during the neonatal period. Consistent with our finding, a similar observation on age-related psychosine increase in white matter was reported in a neuropsychiatric brain cohort between 25 and 68 years of age (Marshall et al., 2018). Three KD brain samples (cases #5, #7, #8) had psychosine levels in the range of 150 to 176 pmol/mg despite differences in age of death. Severity-dependent increases in psychosine levels of 22×, 3.3× and 1.1× (from severe to mild) was observed when normalized to age-matched controls. Given age-dependent increases in psychosine levels in neonates, it is critical to use age-matched controls in measuring psychosine in KD.

In addition to psychosine accumulation, biochemical abnormalities, including increased ASAH1 and hyperglycosylated LAMP1 protein levels, were detected in infantile KD. These increases are likely related to neuroinflammatory response since oligodendrocytes were virtually absent in end-stage KD. Consistent with this notion, hyperglycosylated LAMP1 was localizes mostly in activated microglia of a NPC1-knockout mouse model (Cawley et al., 2020). ASAH1 is a lysosomal cysteine amidase that converts ceramide to sphingosine and its metabolite sphingosine 1-phosphate (Parveen et al., 2019). Deficiency in ASAH1 induces ceramide accumulation, which is a major cause of Farber disease (Sugita et al., 1972). In the context of KD, ASAH1 was recently demonstrated to be a key driver of psychosine accumulation, in part due to the abundance of its substrate, galactosylceramide. Genetic ablation of ASAH1 partially rescues the phenotype of the twitcher mouse model of KD (Li et al., 2019). Here we showed that ASAH1 levels were increased in infantile KD and were mostly expressed in immune cells. It is unclear how psychosine accumulation in immune cells contributes to KD pathogenesis and if ASAH1 drives psychosine accumulation in oligodendrocytes. As more is known about the role of ASAH1 in KD, it will be important to understand the mechanism and extent to which it drives progression of KD.

KD is also known as globoid cell leukodystrophy and is characterized by severe demyelination in central and peripheral nervous systems. Neuroinflammation plays a key role in the pathogenesis of KD. Globoid cells are multinucleated macrophages characterized by accumulation of lipid. We found that astrogliosis paralleled globoid cells accumulation and microgliosis, which were found prior to demyelination. How neuroinflammation contributes to demyelination in KD is unclear. Recently, studies of Schwann cell targeting mice (floxed GALC knockout mice) indicate that normal macrophages attenuate disease by facilitating phagocytosis, but that GALC deficient macrophages promote disease through an inflammatory mechanism (Weinstock et al., 2020). Suppression of astrogliosis by blocking prostaglandin D2 alleviated oligo-dendroglial apoptosis, demyelination and spasticity in twitcher mice, suggesting a pathological role for astrogliosis in KD (Mohri et al., 2006). Our analyses showed increases in globoid cells and astrogliosis in white matter of two infantile KD brains (#5, #7) compared to an age-matched normal brain (case #2). Given the advanced disease stage of both KD cases, there was no difference in levels of demyelination assessed with LFB-PAS staining of myelin or MBP levels with Western blots. On the other hand, globoid cells and astrogliosis increased with disease severity. In particular, more severe pathology was detected in severe KD (case #5), with age of death of 10 months, compared with the milder KD (case #7), with age of death of 21 months. These results strongly suggest that differences in neuropathology are related to disease duration. Case #5 had a disease onset at 4 months of age, consistent with early-infantile KD. Unfortunately, information on symptom-onset was unavailable for case #7. Given that the age of death was 21 months, which is near the high-end for infantile KD (i.e. 24 months), the subject is possibly a late-infantile KD case, which may explain milder neuropathology.

Of note, we detected CD8+, but not CD4+ T lymphocytes in two infantile KD cases, while none were detected in the control case. Consistent with the trend observed for globoid cells and astrogliosis, the number of CD8+ T lymphocytes was three times higher in KD with more severe pathology (case #5) compared to KD with less severe pathology (case #7). Of relevance, both CD4+ and CD8+ T lymphocytes have been reported in demyelinating lesions of multiple sclerosis. CD4+ T lymphocytes are mostly found in acute lesions, whereas the CD8+ T lymphocytes are found in chronic lesions (Raine, 1994). CD4+ T lymphocytes are implicated in the pathogenesis of multiple sclerosis by a strong association of disease susceptibility with MHC class II alleles, but the mechanism is largely unclear (Hafler et al., 2007). Additional similarities between KD and multiple sclerosis include the presence of neuroinflammatory cells (macrophages and astrocytes) associated with demyelinating lesions in both diseases. Recently, *GALC* was identified as a risk factor gene for multiple sclerosis (Li et al., 2021). Immunity-modulating hematopoietic stem cell transplantation is an effective treatment for both diseases (Muraro et al., 2017; Yoon et al., 2021). Fingolimod, an FDA-approved drug for the treatment of multiple sclerosis, was efficacious in improving the phenotype and extending the lifespan of the twitcher mouse model of KD (Bechet et al., 2020). Because of these similarities, understanding pathogenesis and potential treatment options from studies of multiple sclerosis may translate to drug discovery in KD. Given the role of CD8+ and CD4+ T lymphocytes in immune surveillance of white matter (Smolders et al., 2018), one need to be a cautious in interpreting our findings in KD. Further studies of disease progression in KD animal models may clarify the role of CD8+ T lymphocytes in KD.

A limitation of our study is the small number of postmortem brain tissues for the analyses, although we obtained all available cases in public brain banks. The tissue quality also varied; therefore, we compared the immunohistochemical results in select cases. Nevertheless, we could select two infantile cases of different severity and show the correlations between age of death and pathology severity. Further statistical analyses need to be done in larger cohorts.

## 5. Conclusions

Overall, our findings have advanced the understanding of GALC protein biology in the context of normal and KD brain white matter. We also revealed new neuropathological changes that may provide insights to understand KD pathogenesis.

## Supplementary Material

suppl

## Figures and Tables

**Table 1:** Demographics and *GALC* genotypes of the KD and control brain cohort.

Case #	Condition	Symptom Onset Age	Age at Death		Sex	Race	PMI (hours)	*GALC* Genotype		
			Year	Days				Exon #	Allele 1	Allele 2
**1**	Control		0	202	Male	AA	33	15	**p.562Thr**	**p.562Thr**
**2**	Control		1	182	Male		19	15	p.562IIe	**p.562Thr**
**3**	Control		1	263	Male	W	25	1	p.21Ala	**p.21Pro**
								7	p.248Asp	**p.248Asn**
								15	**p.562Thr**	p.562Ile
**4**	Control		40	11	Male		24	15	**p.562Thr**	**p.562Thr**
**5**	Infantile KD	2–3 month	0	294	Male	W	5	1	p.21Ala	**p.21Pro**
								5	**p.184Cys**	p.l84Arg
								7	p.248Asp	**p.248Asn**
								15		p.562Ile
								11–17	***30 kb Del***	
**6**	Infantile KD	NA	1	141	Male	W	4	5	**p.184Cys**	**p.184Cys**
								11–17	***30 kb Del***	***30 kb Del***
**7**	Infantile KD	NA	1	270	Male		29	5	**p.184Cys**	**p.184Cys**
								11–17	***30 kb Del***	***30 kb Del***
**8**	Later-onset KD	13 year	40	118	Male	W	22	1	***p.23X***	p.23Ser
								4	p.117Met	**p.117Leu**
								5	**p.184Cys**	p.184Arg
								15	**p.562Thr**	**p.562Thr**

Amino acid change numbering starts from methionine residue of the first start codon of *GALC* cDNA. Activity reducing polymorphisms and KD-related mutations are highlighted in bold font. Null mutations are highlighted in bold italic font. Abbreviations: AA – African American; W – White; NA – Not available.

## Data Availability

No data was used for the research described in the article.
