# Supplementary material for: Galactosylceramidase deficiency and pathological abnormalities in cerebral white matter of Krabbe disease
Source: Neurobiol Dis. Author manuscript; Available in PMC 2023 Sep 2. (PMC10474820; doi:10.1016/j.nbd.2022.105862)
Supplement: suppl [file NIHMS1917531-supplement-suppl.docx]

**Galactocerebrosidase deficiency, psychosine accumulation and pathological abnormalities in cerebral white matter of Krabbe disease**

Diego Iacono*^1-3^, Shunsuke Koga*^4^, Hui Peng,^1-3^, Arulmani Manavalan^1^, Jessica Daiker^5^,
Monica Castanedes-Casey^4^, Nicholas B. Martin^4^, Aimee R Herdt^1-3^, Michael H. Gelb^5^,
Dennis W. Dickson^4^ and Chris W. Lee^1-3^

*1. Biomedical Research Institute of New Jersey (BRInj), Cedar Knolls, NJ*

*2. Atlantic Health System, Morristown, NJ*

*3. Mid-Atlantic Neonatology Associates (MANA), Morristown, NJ*

*4. Department of Neuroscience, Mayo Clinic Florida, Jacksonville, FL*

*5. Departments of Chemistry and Biochemistry, University of Washington, Seattle, WA*

* These authors equally contributed to the manuscript

**Correspondence to:**

Chris W. Lee, PhD

Biomedical Research Institute of New Jersey (BRInj)

140 East Hanover Avenue

Cedar Knolls, NJ 07927

Email : [chrislee@brinj.org](mailto:chrislee@brinj.org)

**Supplementary Information**

**Supplementary Figure 1. GALC immunoreactivity is seen in oligodendrocytes, but not neurons of control infant brain.** GALC immunoreactivity is seen in oligodendrocytes in various regions of white matter of a control infant (case #2). Magnified views of GALC immunopositivity in the white matter of BA24 (a, b, c), PHG (e, f, g), PVWM (I, j, k) and CC (l, m, n) demonstrate distribution relative to oligodendrocytes. No apparent GALC immunopositivity is detected in neuronal cells of cortex in section of BA24 (d) and PHG (h).

**Supplementary Figure 2. Representative images of globoid cells.** Hematoxylin and eosin (H&E) stain (a, b) and immunohistochemistry for CD68 (c, d) show giant, multinucleated cells in the periventricular white matter of infantile KD case #5.

**
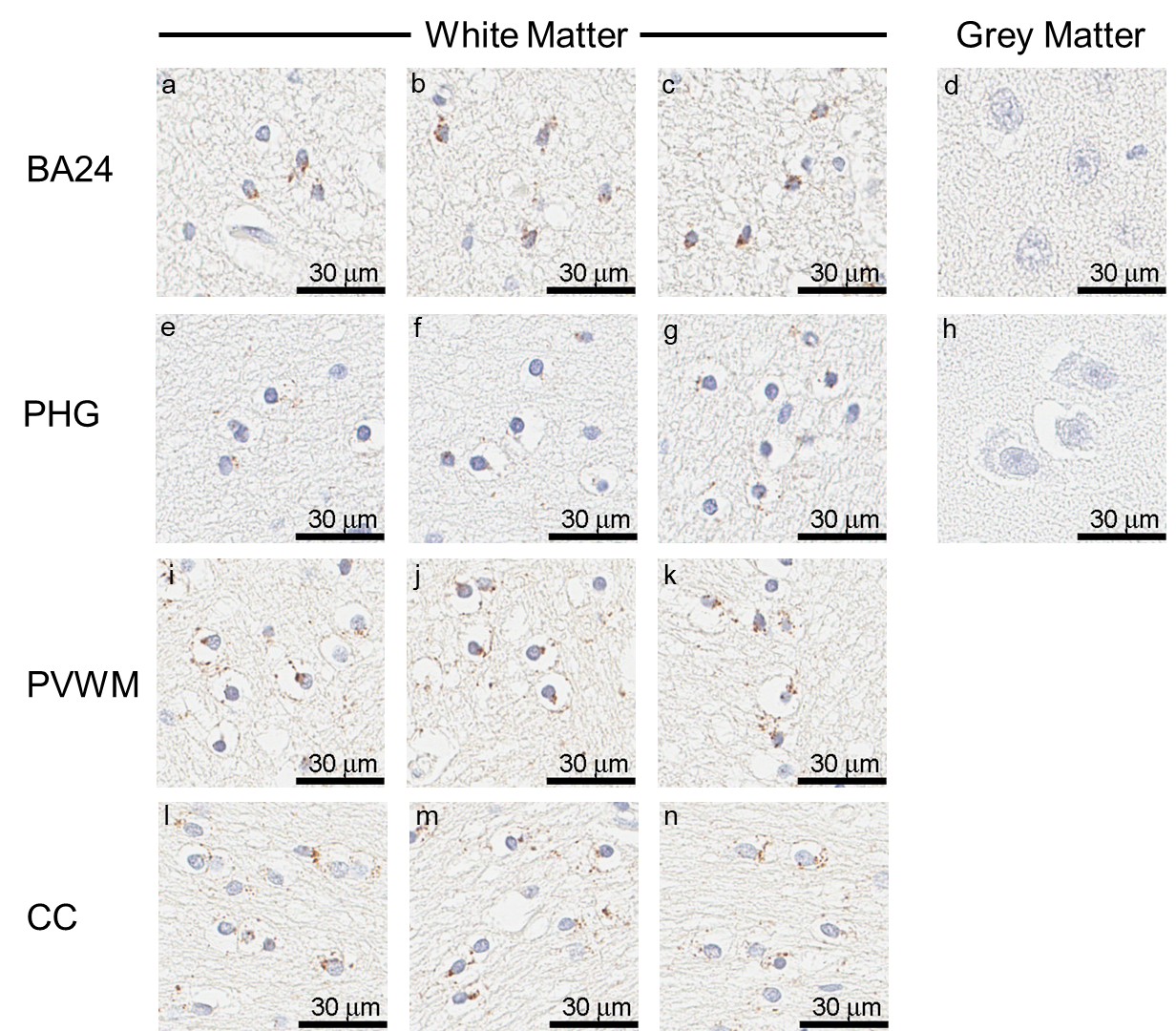
Supplementary Figure 1.** GALC immunoreactivity is seen in oligodendrocytes, but not neurons of control infant brain.

**Supplementary Figure 1. GALC immunoreactivity is seen in oligodendrocytes, but not neurons of control infant brain.** GALC immunoreactivity is seen in oligodendrocytes in various regions of white matter of a control infant (case #2). Magnified views of GALC immunopositivity in the white matter of BA24 (a, b, c), PHG (e, f, g), PVWM (I, j, k) and CC (l, m, n) demonstrate distribution relative to oligodendrocytes. No apparent GALC immunopositivity is detected in neuronal cells of cortex in section of BA24 (d) and PHG (h).

**
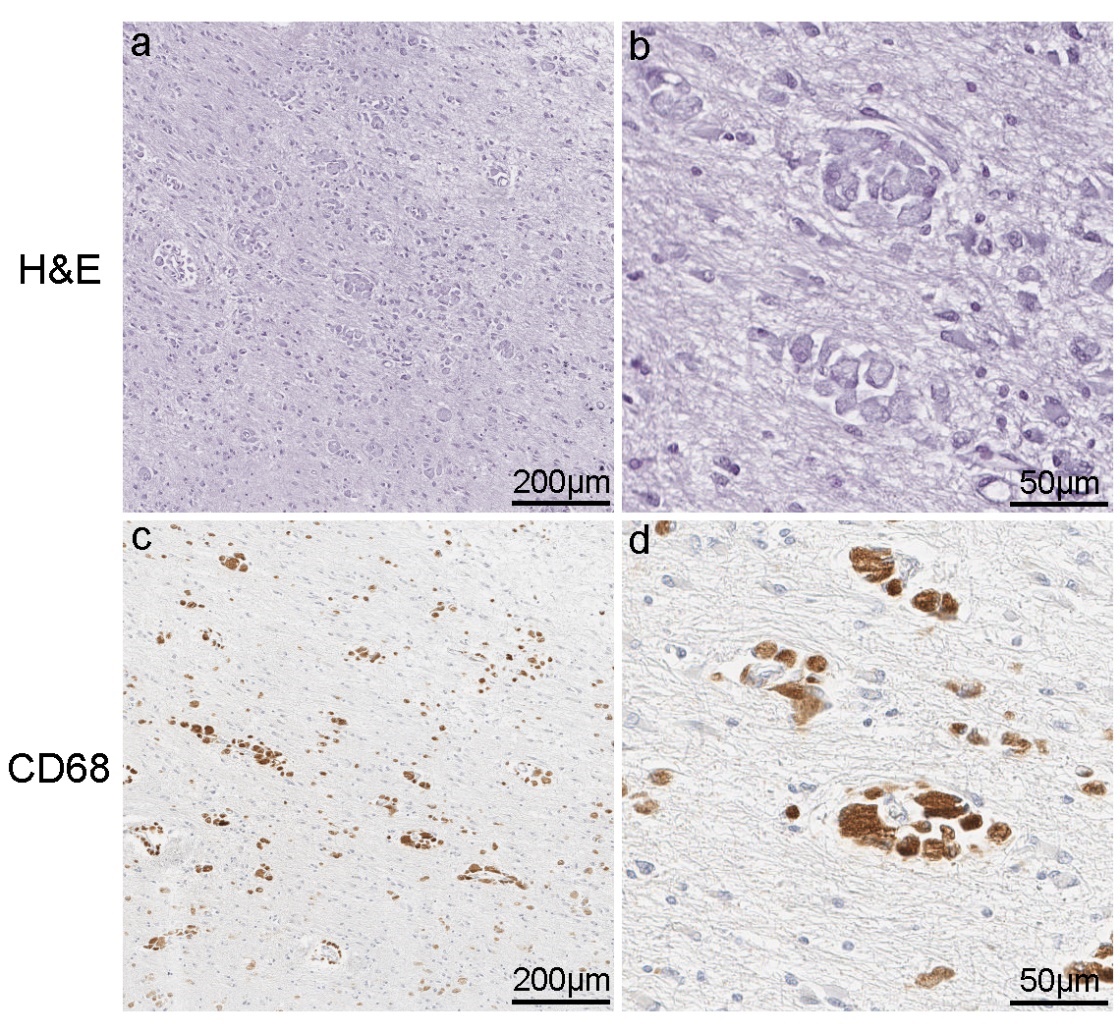
Supplementary Figure 2.** Representative images of globoid cells

**Supplementary Figure 2. Representative images of globoid cells.** Hematoxylin and eosin (H&E) stain (a, b) and immunohistochemistry for CD68 (c, d) show giant, multinucleated cells in the periventricular white matter of infantile KD case #5.
